# Variations in the 3′UTR of the *CYP21A2* Gene in Heterozygous Females with Hyperandrogenaemia

**DOI:** 10.1155/2017/8984365

**Published:** 2017-04-12

**Authors:** Vassos Neocleous, Pavlos Fanis, Meropi Toumba, Alexia A. P. Phedonos, Michalis Picolos, Elena Andreou, Tassos C. Kyriakides, George A. Tanteles, Christos Shammas, Leonidas A. Phylactou, Nicos Skordis

**Affiliations:** ^1^Department of Molecular Genetics, Function & Therapy, The Cyprus Institute of Neurology & Genetics, Nicosia, Cyprus; ^2^Pediatric Endocrine Clinic, IASIS Hospital, Paphos, Cyprus; ^3^Alithias Endocrinology Center, Nicosia, Cyprus; ^4^Dasoupolis Endocrinology Center, Andrea Dimitriou Street Dasoupolis, Nicosia, Cyprus; ^5^Yale Center for Analytical Sciences, Yale School of Public Health, New Haven, CT, USA; ^6^Clinical Genetics Department, The Cyprus Institute of Neurology & Genetics, Nicosia, Cyprus; ^7^Division of Pediatric Endocrinology, Paedi Center for Specialized Pediatrics, Nicosia, Cyprus; ^8^St. George's University of London Medical School at the University of Nicosia, Nicosia, Cyprus

## Abstract

Heterozygosity for *CYP21A2* mutations in females is possibly related to increased risk of developing clinical hyperandrogenism. The present study was designed to seek evidence on the phenotype-genotype correlation in female children, adolescents, and women with *CYP21A2* mutations and variants in the 3′UTR region of the gene. Sixty-six patients out of the 169 were identified as carriers of *CYP21A2* mutations. Higher values of stimulated 17 hydroxyprogesterone (17-OHP) levels were found in the carriers of the p.Val281Leu mutation compared to the carriers of other mutations (mean: 24.7 nmol/l versus 15.6 nmol/l). The haplotype of the ^∗^52C>T, ^∗^440C>T, and ^∗^443T>C in the 3′UTR was identical in all heterozygous patients with p.Val281Leu and the haplotype of the ^∗^12C>T and ^∗^52C>T was identical in all heterozygous patients with the p.Gln318^∗^. In conclusion, hyperandrogenaemic females are likely to bear heterozygous *CYP21A2* mutations. Carriers of the mild p.Val281Leu mutation are at higher risk of developing hyperandrogenism than the carriers of more severe mutations. The identification of variants in the 3′UTR of *CYP21A2* in combination with the heterozygous mutation may be associated with the mild form of nonclassic congenital adrenal hyperplasia and reveal the importance of analyzing the *CYP21A2* untranslated regions for the appropriate management of this category of patients.

## 1. Introduction

Congenital adrenal hyperplasia (CAH) is an autosomal recessive disorder caused by mutations in one of the genes involved in steroidogenesis. More than 95% of all cases of CAH are due to 21-hydroxylase deficiency (21-OHD) resulting from defects in the steroid 21-hydroxylase (*CYP21A2*) gene. CAH is classified into the severe classical form with an overall estimated incidence of 1 : 10,000 to 1 : 15,000 and into the mild nonclassical form (NC-CAH) ranging from 1 : 500 to 1 : 100 live births [1–3].

Since the prevalence of CAH and NC-CAH has been reported to vary among populations, the expected heterozygote frequency for 21-OHD also varies considerably and ranges from 1 : 10 to 1 : 60 in specific populations [4–8]. Moreover, for certain ethnic groups such as the Ashkenazi Jews, the carrier frequency was reported to be 1 : 3, thus making it to be the highest ever reported for the disease [9]. To date, the occurrence of genetic mutants of 21-OHD has overwhelmingly been investigated and ethnic detailed distribution of mutations has been described [10–20]. Roughly 95% of the *CYP21A2* alleles bearing mutations are due to recombination events among the homologous inactive *CYP21A2P* pseudogene and the *CYP21A2* gene. The remaining 5% of the *CYP21A2* mutations is the result of new casual mutagenic events [21, 22]. It should be noted though that an estimated 20% of the alleles in the NC-CAH form remain without identified causative mutations, thus signifying the necessity to investigate the *CYP21A2* regulatory regions [23].

At this time, numerous studies have implicated a number of variations in the promoter and the 5′UTR of the *CYP21A2* [24–26]. Recently, in vitro and bioinformatics analysis for the sequence variation ^∗^13G>A in the 3′UTR predicted a modification of the RNA expression and folding was found to correlate with a mild form of NC-CAH [27]. The 3′UTR plays a vital role in gene expression by regulating the localization, export, stability, and translational competence of an mRNA. Variations in the 3′UTR might change the mRNA secondary structure and are identified to be accountable for human diseases [28]. In population studies with a large number of nonclassical patients, the percentage of alleles with recognised mutations is variable, ranging from 80% to 100% [29–32], signifying the stipulation to evaluate the *CYP21A2* regulatory regions.

Numerous studies in the Mediterranean Basin, including research from our group, have confirmed as the most predominant genetic defects, the mutations IVS2-13A/C>G, p.Gln318^∗^, p.Val281Leu, and c.329_336del (8bpdelE3) [12, 13, 33, 34]. Compared to normal female individuals, female carriers for 21-OHD frequently demonstrate an increased secretion of the 21-OH precursors 17-hydroxyprogesterone (17-OHP) and progesterone (P4) [35–43] and lower levels of 11-deoxycorticosterone [37] as expected. Furthermore in obligate heterozygotes, patients for the simple virilizing form of CAH, aldosterone secretion was found to be reduced following ACTH stimulation, suggesting an impairment of the *zona glomerulosa* in addition to *zona fasciculata* [44].

Several studies from various groups have conveyed that between 50 and 80% of carriers exhibit a 17-OHP level after ACTH stimulation and that is above the 95th percentile of the control value [37, 39, 42, 45]. The purpose of this study was to determine the frequency of identified defects of the *CYP21A2* gene and also to weigh the regulatory 3′UTR region of the gene in a clinically symptomatic cohort of 169 females, characterized by hyperandrogenaemia. To test this hypothesis, the total of 169 patients was tested by Sanger DNA sequencing and MLPA analysis for defects in the *CYP21A2* gene.

## 2. Subjects and Methods

### 2.1. Biochemical and Clinical Evaluation

A total of 169 unrelated Greek Cypriot females were diagnosed and 47 were children, 30 adolescents, and 92 women. The study was approved by the Cyprus National Bioethics Committee and written consent was obtained from all adult patients and the parents or guardians of the minors. The diagnosis of the patients was based on the clinical findings and elevated stimulated plasma 17-OHP [1, 10]. A standard dose of ACTH stimulation test was used. Measurements of the basal and after 60 minutes ACTH administration serum 17-OHP concentrations were obtained with the commercial RIA method (Beckman Coulter).

The patients of the study were clinically evaluated based on hyperandrogenaemia symptoms. These symptoms included early appearance of pubic or axillary hair (before the age of 8 years), severe acne or hirsutism (determined by a Ferriman-Gallwey score of >8) in adolescents and adults, abnormal menstrual cycles with or without polycystic ovaries, and complete absence of virilisation.

### 2.2. Statistical Analysis

Comparisons of 17-OHP levels were accomplished using parametric methods (*t*-test procedures). Frequency distributions of the 3′UTR and 5′UTR/promoter variants of the *CYP212A2* gene were tabulated and compared using chi-square methods. A two-tailed alpha level of 0.05 was used to establish statistical significance. All statistical analyses were done using SAS software Version 9.2, SAS Institute Inc., Cary, NC, USA. (i) The results of the 17-OHP levels are shown in the text and expressed as mean ± SD. (ii) The frequency distribution of the 3′UTR and the 5′UTR/promoter variants of the *CYP21A2* gene was estimated using the FREQ procedure.

### 2.3. Amplification of the *CYP21A2* Gene

The *CYP21A2* gene of the total number of patients who participated in the study was analyzed by Sanger DNA sequencing. More specifically, the *CYP21A2* promoter/regulatory regions from the patients participating in this study and 150 normal females were analyzed in this paper. The genetic investigation was done based on a cascade strategy as formerly described [12, 13, 33]. For the amplification of the 5′UTR region that is located in the first 167 nucleotides upstream the ATG codon of the *CYP21A2* gene, the primers P1–P48 [46] were used to amplify a fragment of 370 bp. The 3′UTR region that is 536 nucleotides downstream the TGA stop codon of the *CYP21A2* gene was amplified using the primers: 5′AGATGCAGCCTTTCCAAGTG3′ and 5′AGCACAGTGGACCATCAGGT3′ [27].

### 2.4. MLPA Analysis

The multiplex ligation-dependent probe amplification (MLPA) technique (MRC Holland, Amsterdam, Netherlands) was used to detect any possible large gene deletions, duplications, and large gene conversions in the *CYP21A2* gene. DNA from the 169 female patients in this study analyzed by direct sequencing was also examined with MLPA [13].

### 2.5. Secondary Structure Analysis

The 2182 bp complete mRNA, including the 5′UTR, coding region, and 3′UTR, was submitted to the RNAfold WebServer (http://rna.tbi.univie.ac.at/cgi-bin/RNAfold.cgi) with default parameters to predict the potential secondary structure. Secondary structures were predicted for the wild-type, the 281L/^∗^52/^∗^440/^∗^443 mutant, and the 318Ter/^∗^12/^∗^52 mutant *CYP21A2* mRNA. Potential minimum free energy (MFE) structures, centroid structures, and positional entropies were obtained [47].

## 3. Results

### 3.1. Clinical and Laboratory Characteristics

In a total of 169 unrelated hyperandrogenaemic females, 66 were identified as carriers of *CYP21A2* mutations (Table 1). Twenty-one of the *CYP21A2* heterozygote female patients were presented in childhood with premature adrenarche (age 3–10 years). The remaining 45 *CYP21A2* heterozygote female patients were diagnosed in adolescence (14–17 years) and adulthood (18–35 years) with clinical signs of hyperandrogenaemia. Among the 45 adolescent and adult *CYP21A2* heterozygote female patients, the most common presenting symptom was irregular menses with or without multicystic ovaries on the ultrasound (32/45), followed by hirsutism in 21/45.

ACTH stimulation test was performed on 104 females (52 carriers and 52 noncarriers). Mean plasma basal 17-OHP (nmol/l) level in the noncarriers was 5.9 ± 1.9 (mean ± SD, range 10.2–2.2) and rose to 12.6 ± 3.8 (mean ± SD, range 19.4–7.0) after ACTH stimulation. Carriers demonstrated a higher mean plasma basal 17-OHP level (nmol/l) 6.5 ± 3.5 (mean ± SD, range 17.6–1.7) and after ACTH stimulation rose to 19.0 ± 12.6 (mean ± SD, range 30.2–1.4) (*p* < 0.0003). The hormonal analyses for 17-OHP were performed in 52 patients.

Two of these patients with p.Val281Leu/X which were hormonally evaluated had stimulated 17-OHP levels more than 60.5 nmol/l and were excluded from the statistical evaluation. Therefore, the implication of digenic inheritance in the above patient might be the case for the development of CAH.

### 3.2. *CYP21A2* Genotypic Analysis

The most frequent mutation within the 66 unrelated alleles was p.Val281Leu (53.0%), followed by p.Gln318^∗^ (18.2%), p.Pro482Ser (10.6%), p.Val304Met (6.1%), p.Pro453Ser (6.1%), p.Ala391Thr (1.5%), large deletion/conversion exons 1–4 (1.5%), and microconversion of exons 6–8 and 8bpdelE3 (1.5%). MLPA results confirm that the carriers with the p.Gln318^∗^ are real and exclude the possibility of false positives due to influence of the pseudogene duplications. Phenotype-genotype correlation of the carrier hyperandrogenaemic females is presented in Table 1. Higher mean plasma values for 17-OHP after ACTH stimulation were exhibited in female carriers of the p.Val281Leu mutation when compared to the mean values observed in female carriers of other *CYP21A2* mutations (24.7 nmol/l versus 15.6 nmol/l) (*p* < 0.0001).

The remaining 103 females who manifested clinical signs of hyperandrogenaemia and were identified with no mutation in the *CYP21A2* gene exhibited an unusual high allelic frequency of 56.3% for the p.Asn493Ser variant. On the contrary, the allelic frequency of the p.Asn493Ser variant was significantly different when compared to the one observed in the group of the 66 *CYP21A2* heterozygous females (56.3% versus 19.7%, *p* < 0.0001).

### 3.3. Extended Sequencing of the Promoter/5′UTR and the 3′UTR of the *CYP21A2*

The aim of the study was to identify 3′UTR and 5′UTR variations in patients presented with clinical signs with hyperandrogenaemia who were found to be heterozygotes for the *CYP21A2* gene. Therefore, these variations were not tested in patients with both forms of 21-OHD carrying two affected alleles.

Extended sequencing of the 3′UTR of the *CYP21A2* in 66 female patients of the present study identified with one affected *CYP21A2* allele demonstrated that 29/35 female patients who carried in heterozygosity the missense mutation p.Val281Leu also carry in the 3′UTR the variants ^∗^52T>C, ^∗^440C>T, and ^∗^443T>C in *cis*. In a similar fashion, 9/12 of the other heterozygote females with the severe p.Gln318^∗^ mutation were identified to also carry in the 3′UTR the variants ^∗^12T>C and ^∗^52C>T in *cis* (Table 2). For the children and adolescents that participated in the study, the parental samples were available and segregation analysis determined and proved that p.Val281Leu mutation and variations ^∗^52C>T, ^∗^440C>T, and ^∗^443T>C are in *cis*. For the rest of the women that participated in the study, no parental samples were available. Sequencing analysis of the *CYP21A2* 3′UTR of a cohort of 90 hyperandrogenaemic females with no detected mutations in the *CYP21A2* gene identified both the combination of 3′UTR variants ^∗^52C>T, ^∗^440C>T, and ^∗^443T>C and ^∗^12T>C and ^∗^52C>T only once (1.1%) in two different patients (Table 2).

Concurrent screening of the *CYP21A2* 3′UTR in 150 control females with no hyperandrogenism and no detected mutations in the *CYP21A2* gene identified the combination of 3′UTR variants ^∗^52C>T, ^∗^440C>T, and ^∗^443T>C in 12/150 (8%) and ^∗^12C>T and ^∗^52C>T in 8/150 (5.3%) (Table 2).

The extended sequencing of the *CYP21A2* promoter/5′UTR region in all groups of patients and controls mentioned above did not reveal any unusual variants or frequencies.

### 3.4. *CYP21A2* mRNA Secondary Structure Prediction

**Figure 1:**
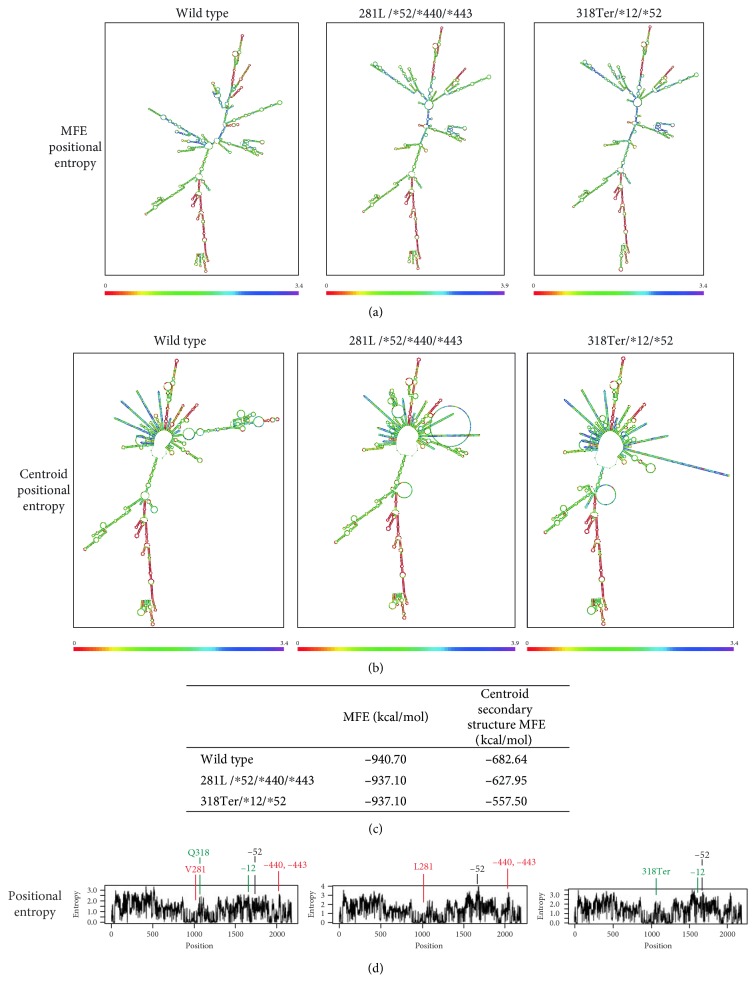
*CYP21A2* mRNA secondary structure prediction. (a) and (b) predicted *CYP21A2* mRNA secondary structures showing the MFE positional and centroid positional entropy, respectively, for the wild type, 281L/^∗^52/^∗^440/^∗^443 mutant, and 318Ter/^∗^12/^∗^52 mutant. (c) Minimum free energy (MFE) and centroid secondary structure MFE values. mRNA secondary structures values with 1 kcal/mol above the minimum MFE observed in wild type are considered responsible for the destabilisation of the structure [48]. (d) Positional entropies for the wild type, 281L/^∗^52/^∗^440/^∗^443 mutant, and 318Ter/^∗^12/^∗^52 mutant. Locations of the variations are indicated. 281L/^∗^52/^∗^440/^∗^443 mutant variations are indicated with red colour. 318Ter/^∗^12/^∗^52 mutant variations are indicated with green colour. ^∗^52 variation which is common in the two mutants is indicated with black colour.

Secondary structure prediction analysis of the wild-type *CYP21A2* mRNA was compared to the 281L/^∗^52/^∗^440/^∗^443 and the 318Ter/^∗^12/^∗^52 variant mRNAs that are displayed in Figures 1(a) and 1(b). In those figures, the MFE and the centroid positional entropy MFE values are, respectively, displayed and more structural changes caused by the *CYP21A2* variants were observed between the two forms. Moreover, the MFE and the centroid secondary structure MFE values of the predicted mRNA secondary structures were also found to be higher in the 281L/^∗^52/^∗^440/^∗^443 and 318Ter/^∗^12/^∗^52 variants when compared to the wild-type form. This finding could be attributed to a possible reduced stability of the secondary mRNA structure (Figure 1(c)). Furthermore, position entropy for the variation located at the ^∗^52 position in the 3′UTR is higher compared to wild-type *CYP21A2* mRNA which might be responsible for destabilizing the mRNA secondary structure (Figure 1(d)).

## 4. Discussion

Up to date, a large spectrum of mutations in the *CYP21A2* gene has been reported. Most of these reported mutations affect the coding region of the gene and to a lesser extent the introns and the promoter [27]. Interestingly, variants located in the 3′ UTR of *CYP21A2* which among other regulatory elements contain several microRNA-binding sites have not yet been reported to be associated with CAH. As 3′UTR mutations can influence the disease susceptibility by altering protein and microRNA- (miRNA-) binding regions, we screened the *CYP21A2* 3′UTR for mutations in the *CYP21A2* heterozygote hyperandrogenaemic females and the hyperandrogenaemic females with no mutations. Among the variations lying at the promoter and the 3′UTR noncoding regions, the combination of 3′UTR variants ^∗^52C>T, ^∗^440C>T, ^∗^443T>C, ^∗^12C>T, and ^∗^52C>T were present in a statistically significant number of *CYP21A2* heterozygous females with the mild NC-CAH phenotype. Interestingly, the variants ^∗^52C>T, ^∗^440C>T, and ^∗^443T>C were present in all heterozygous females identified with the p.Val281Leu missense mutation. In a similar fashion, the variants ^∗^12C>T and ^∗^52C>T were present also in all females identified with the p.Gln318^∗^ mutation.

On the contrary, the distribution of both ^∗^52C>T, ^∗^440C>T, ^∗^443T>C and ^∗^12C>T, ^∗^52C>T variants was not observed in any of the other *CYP21A2* heterozygote females of the present study. Twelve known *CYP21A2* 3′UTR variants were identified, the majority of which were unique to the three different examined cohorts. Additionally, the distribution of both ^∗^52C>T, ^∗^440C>T, ^∗^443T>C and ^∗^12C>T, ^∗^52C>T in the cohort of the sex-matched female controls and the cohort of hyperandrogenaemic females identified with no mutation in the *CYP21A2* gene were very low and almost negligible.

Secondary structure for *CYP21A2* mRNA molecule was predicted using the RNAfold WebServer, which provides the thermodynamically favoured structure. The mRNA secondary structure is a critical characteristic in the function of *cis*-regulatory elements located in the 3′UTR. Comparison of the *CYP21A2* wild-type, 281L/^∗^52/^∗^440/^∗^443 mutant, and 318Ter/^∗^12/^∗^52 mutant mRNA secondary structures showed distinct differences. The higher MFE of the mutant mRNA secondary structures and the distinct higher position entropy of the ^∗^52 variation at the 3′UTR indicate less stable mRNA secondary structure which might contribute to the clinical manifestation of the disease. However, mRNA structure differences cannot be proposed as a single factor responsible for a disease.

In conclusion, females with hyperandrogenism are likely to bear heterozygous *CYP21A2* mutations. The identification of variants in the 3′UTR of the *CYP21A2* gene in combination with the heterozygous mutation may be associated with the mild form of the disease and reveal the importance of analyzing the *CYP21A2* untranslated regions to better characterize and treat this category of patients. Insights obtained from studies that identify the genetic basis of detrimental disorders such as CAH are particularly useful since they can provide a better understanding of disease pathogenesis, used for more effective diagnostic confirmation, assist in genetic counselling, and used in the development of newer therapeutic methodologies.

## Figures and Tables

**Table 1:** Phenotype-genotype correlation of the 66 females with *CYP21A2* heterozygote mutations. The hormonal analyses for 17-OHP were performed in 52 patients. Two of the patients with p.Val281Leu/X had stimulated 17-OHP levels more than 60.5 nmol/l viewing the possibility of an additional unidentified mutation and were excluded from the statistical evaluation.

	Phenotype	Genotype								
		p.Val281Leu/X	p.Gln318^∗^/X	p.Pro482Ser/X	p.Val304Met/X	p.Pro453Ser/X	p.Ala391Thr/X	Large Del 1_4	Del EX 6_8	8bpdeE3/X
Children	*N* = 19	12	2	—	1	2	—	1	1	—
	Basal 17-OHP (nmol/l) *N* = 16	2.2–7.7	15.6		8.3	2.2		2.4		
	Stimulated 17-OHP (nmol/l) *N* = 16	20–24.2	27		13.7	32		14.5		

Adolescents	*N* = 17	8	5	2	2	—	—	—	—	—
	Basal 17-OHP (nmol/l) *N* = 13	2.6–12.4	4.2–9.2	3.5–6.7	2.4–5.1					
	Stimulated 17-OHP (nmol/l) *N* = 13	11–28	10.3–19.6	14.2–17.1	9.6–4.8					

Adults	*N* = 30	15	5	5	1	2	1	—	—	1
	Basal 17-OHP (nmol/l) *N* = 23	3.4–14.2	4.6–17.6	1.7–8.9	5.6	4.4	6.1			8.8
	Stimulated 17-OHP (nmol/l) *N* = 23	11–30.2	1.4–18.1	8.9–14.7	12	21.1	15.8			12.3

**Table 2:** Haplotypes of the alleles with the variants found in the 3′UTR of the *CYP21A2* gene.

Haplotypes	Female controls (*n* = 150)	Female controls (%)	Hyperandrogenic females with no mutation (*n* = 90)	Hyperandrogenic females with no mutation (%)	*CYP21A2* heterozygotes (*n* = 66)	*CYP21A2* heterozygotes (%)
0	62	41.3	55	61.1	8	12.1
1	12	8.0	1	1.1	29^¥^	43.9
2	28	18.7	16	17.8	11	16.7
3	21	14.0	8	8.8	0	0
4	8	5.3	1	1.1	9^§^	13.7
5	6	4.0	0	0	3	4.5
6	9	6.0	4	4.4	3	4.5
7	4	2.7	3	3.3	0	0
8	0	0	2	2.2	3	4.5

0 = no variants; 1 = ^∗^52C>T, 440C>T, 443T>C; 2 = ^∗^52C>T; 3 = ^∗^368T>C, 390A>G, 440C>T, 443T>C, 464T>C, 474C>T; 4 = ^∗^12C>T, 52C>T; 5 = ^∗^52C>T, 368T>C, 390A>G, 440C>T, 443T>C, 464T>C, 474C>T; 6 = ^∗^176C>T; 7 = ^∗^13G>A; 8 = ^∗^93G>A, 368T>C, 390A>G, 440C>T, 443T>C, 464T>C, 474C>T, 496C>T.

^¥^All 29 females were heterozygote for p.Val281Leu, and all shared the same ^∗^52C>T, 440C>T, and 443T>C combination of variants.

^§^All 9 females were heterozygote for p.Gln318^∗^, and all shared the same ^∗^12C>T and 52C>T combination of variants.

## References

[1] Speiser P. W., White P. C. (2003). Congenital adrenal hyperplasia. *The New England Journal of Medicine*.

[2] Carroll M. C., Campbell R. D., Porter R. R. (1985). Mapping of steroid 21-hydroxylase genes adjacent to complement component C4 genes in HLA, the major histocompatibility complex in man. *Proceedings of the National Academy of Sciences of the United States of America*.

[3] Merke D. P., Bornstein S. R. (2005). Congenital adrenal hyperplasia. *Lancet*.

[4] New M. I., Lorenzen F., Lerner A. J. (1983). Genotyping steroid 21-hydroxylase deficiency: hormonal reference data. *The Journal of Clinical Endocrinology and Metabolism*.

[5] Speiser P. W., Dupont B., Rubinstein P., Piazza A., Kastelan A., New M. I. (1985). High frequency of nonclassical steroid 21-hydroxylase deficiency. *American Journal of Human Genetics*.

[6] Brodie B. L., Wentz A. C. (1987). Late onset congenital adrenal hyperplasia: a gynecologist's perspective. *Fertility and Sterility*.

[7] Baumgartner-Parzer S. M., Nowotny P., Heinze G., Waldhausl W., Vierhapper H. (2005). Carrier frequency of congenital adrenal hyperplasia (21-hydroxylase deficiency) in a middle European population. *The Journal of Clinical Endocrinology and Metabolism*.

[8] Phedonos A. A., Shammas C., Skordis N., Kyriakides T. C., Neocleous V., Phylactou L. A. (2013). High carrier frequency of 21-hydroxylase deficiency in Cyprus. *Clinical Genetics*.

[9] White P. C., New M. I., Dupont B. (1986). Structure of human steroid 21-hydroxylase genes. *Proceedings of the National Academy of Sciences of the United States of America*.

[10] Dracopoulou-Vabouli M., Maniati-Christidi M., Dacou-Voutetakis C. (2001). The spectrum of molecular defects of the CYP21 gene in the Hellenic population: variable concordance between genotype and phenotype in the different forms of congenital adrenal hyperplasia. *The Journal of Clinical Endocrinology and Metabolism*.

[11] Wilson R. C., Nimkarn S., Dumic M. (2007). Ethnic-specific distribution of mutations in 716 patients with congenital adrenal hyperplasia owing to 21-hydroxylase deficiency. *Molecular Genetics and Metabolism*.

[12] Skordis N., Shammas C., Efstathiou E., Kaffe K., Neocleous V., Phylactou L. A. (2011). Endocrine profile and phenotype-genotype correlation in unrelated patients with non-classical congenital adrenal hyperplasia. *Clinical Biochemistry*.

[13] Skordis N., Kyriakou A., Tardy V. (2011). Molecular defects of the CYP21A2 gene in Greek-Cypriot patients with congenital adrenal hyperplasia. *Hormone Research in Pædiatrics*.

[14] Bidet M., Bellanne-Chantelot C., Galand-Portier M. B. (2009). Clinical and molecular characterization of a cohort of 161 unrelated women with nonclassical congenital adrenal hyperplasia due to 21-hydroxylase deficiency and 330 family members. *The Journal of Clinical Endocrinology and Metabolism*.

[15] Dolzan V., Solyom J., Fekete G. (2005). Mutational spectrum of steroid 21-hydroxylase and the genotype-phenotype association in middle European patients with congenital adrenal hyperplasia. *European Journal of Endocrinology*.

[16] Ezquieta B., Oliver A., Gracia R., Gancedo P. G. (1995). Analysis of steroid 21-hydroxylase gene mutations in the Spanish population. *Human Genetics*.

[17] Abid F., Tardy V., Gaouzi A., El Hessni A., Morel Y., Chabraoui L. (2008). CYP21A2 gene mutation analysis in Moroccan patients with classic form of 21-hydroxylase deficiency: high regional prevalence of p.Q318X mutation and identification of a novel p.L353R mutation. *Clinical Chemistry and Laboratory Medicine*.

[18] Krone N., Braun A., Roscher A. A., Knorr D., Schwarz H. P. (2000). Predicting phenotype in steroid 21-hydroxylase deficiency? Comprehensive genotyping in 155 unrelated, well defined patients from southern Germany. *The Journal of Clinical Endocrinology and Metabolism*.

[19] Wedell A., Thilen A., Ritzen E. M., Stengler B., Luthman H. (1994). Mutational spectrum of the steroid 21-hydroxylase gene in Sweden: implications for genetic diagnosis and association with disease manifestation. *The Journal of Clinical Endocrinology and Metabolism*.

[20] Carvalho D. F., Miranda M. C., Gomes L. G. (2016). Molecular CYP21A2 diagnosis in 480 Brazilian patients with congenital adrenal hyperplasia before newborn screening introduction. *European Journal of Endocrinology*.

[21] Higashi Y., Tanae A., Inoue H., Fujii-Kuriyama Y. (1988). Evidence for frequent gene conversion in the steroid 21-hydroxylase P-450(C21) gene: implications for steroid 21-hydroxylase deficiency. *American Journal of Human Genetics*.

[22] Donohoue P. A., Jospe N., Migeon C. J., Van Dop C. (1989). Two distinct areas of unequal crossingover within the steroid 21-hydroxylase genes produce absence of CYP21B. *Genomics*.

[23] Neocleous V., Phedonos A. A. P., Picolos M. (2013). Coding mutations and variations in the 3′UTR of CYP21A2 gene in heterozygous females associate with hyperandrogenism. http://www.ashg.org/2013meeting/abstracts/fulltext/f130120023.htm.

[24] Araujo R. S., Mendonca B. B., Barbosa A. S. (2007). Microconversion between CYP21A2 and CYP21A1P promoter regions causes the nonclassical form of 21-hydroxylase deficiency. *The Journal of Clinical Endocrinology and Metabolism*.

[25] Concolino P., Mello E., Minucci A. (2009). A new CYP21A1P/CYP21A2 chimeric gene identified in an Italian woman suffering from classical congenital adrenal hyperplasia form. *BMC Medical Genetics*.

[26] Zhang H. J., Yang J., Zhang M. N. (2009). Variations in the promoter of CYP21A2 gene identified in a Chinese patient with simple virilizing form of 21-hydroxylase deficiency. *Clinical Endocrinology*.

[27] Menabo S., Balsamo A., Baldazzi L. (2012). A sequence variation in 3′UTR of CYP21A2 gene correlates with a mild form of congenital adrenal hyperplasia. *Journal of Endocrinological Investigation*.

[28] Chatterjee S., Pal J. K. (2009). Role of 5'- and 3'-untranslated regions of mRNAs in human diseases. *Biology of the Cell*.

[29] Deneux C., Tardy V., Dib A. (2001). Phenotype-genotype correlation in 56 women with nonclassical congenital adrenal hyperplasia due to 21-hydroxylase deficiency. *The Journal of Clinical Endocrinology and Metabolism*.

[30] Barbat B., Bogyo A., Raux-Demay M. C. (1995). Screening of CYP21 gene mutations in 129 French patients affected by steroid 21-hydroxylase deficiency. *Human Mutation*.

[31] Carrera P., Bordone L., Azzani T. (1996). Point mutations in Italian patients with classic, non-classic, and cryptic forms of steroid 21-hydroxylase deficiency. *Human Genetics*.

[32] Weintrob N., Brautbar C., Pertzelan A. (2000). Genotype-phenotype associations in non-classical steroid 21-hydroxylase deficiency. *European Journal of Endocrinology*.

[33] Neocleous V., Ioannou Y. S., Bartsota M., Costi C., Skordis N., Phylactou L. A. (2009). Rare mutations in the CYP21A2 gene detected in congenital adrenal hyperplasia. *Clinical Biochemistry*.

[34] Skordis N., Shammas C., Phedonos A. A. (2015). Genetic defects of the CYP21A2 gene in girls with premature adrenarche. *Journal of Endocrinological Investigation*.

[35] Neocleous V., Shammas C., Phedonos A. A., Phylactou L. A., Skordis N. (2014). Phenotypic variability of hyperandrogenemia in females heterozygous for CYP21A2 mutations. *Indian Journal of Endocrinology Metabolism*.

[36] Neocleous V., Shammas C., Phedonos A. P. (2012). Genetic defects in the cyp21a2 gene in heterozygous girls with premature adrenarche and adolescent females with hyperandrogenemia. *Georgian Medical News*.

[37] Peter M., Sippell W. G., Lorenzen F., Willig R. P., Westphal E., Grosse-Wilde H. (1990). Improved test to identify heterozygotes for congenital adrenal hyperplasia without index case examination. *Lancet*.

[38] Krensky A. M., Bongiovanni A. M., Marino J., Parks J., Tenore A. (1977). Identification of heterozygote carriers of congenital adrenal hyperplasia by radioimmunoassay of serum 17-OH progesterone. *The Journal of Pediatrics*.

[39] Gutai J. P., Kowarski A. A., Migeon C. J. (1977). The detection of the heterozygous carrier for congenital virilizing adrenal hyperplasia. *The Journal of Pediatrics*.

[40] Weil J., Bidlingmaier F., Sippell W. G., Butenandt O., Knorr D. (1979). Comparison of two tests for heterozygosity in congenital adrenal hyperplasia (CAH). *Acta Endocrinologica*.

[41] Lee P. A., Gareis J. F. (1975). Evidence for partial 21-hydroxylase deficiency among heterozygote carriers of congenital adrenal hyperplasia. *The Journal of Clinical Endocrinology and Metabolism*.

[42] Handelsman D. J., Howe C. J., Conway A. J., Turtle J. R. (1983). Heterozygote detection in congenital adrenal hyperplasia. *Clinical Chemistry*.

[43] Nemeth S., Riedl S., Kriegshauser G. (2012). Reverse-hybridization assay for rapid detection of common CYP21A2 mutations in dried blood spots from newborns with elevated 17-OH progesterone. *Clinica Chimica Acta*.

[44] Pardini D. P., Kater C. E., Vieira J. G., Biglieri E. G. (1983). Impaired mineralocorticoid hormone responses to adrenocorticotropin stimulation: additional characterization of heterozygosity for the 21-hydroxylase deficiency type of congenital adrenal hyperplasia. *The Journal of Clinical Endocrinology and Metabolism*.

[45] Petersen K. E., Svejgaard A., Nielsen M. D., Dissing J. (1982). Heterozygotes and cryptic patients in families of patients with congenital adrenal hyperplasia (21-hydroxylase deficiency). HLA and glyoxalase I typing and hormonal studies. *Hormone Research*.

[46] Wedell A., Luthman H. (1993). Steroid 21-hydroxylase deficiency: two additional mutations in salt-wasting disease and rapid screening of disease-causing mutations. *Human Molecular Genetics*.

[47] Gruber A. R., Lorenz R., Bernhart S. H., Neubock R., Hofacker I. L. (2008). The Vienna RNA websuite. *Nucleic Acids Research*.

[48] Shu W., Bo X., Liu R., Zhao D., Zheng Z., Wang S. (2006). RDMAS: a web server for RNA deleterious mutation analysis. *BMC Bioinformatics*.

